# Clonal growth strategy, diversity and structure: A spatiotemporal response to sedimentation in tropical *Cyperus papyrus* swamps

**DOI:** 10.1371/journal.pone.0190810

**Published:** 2018-01-16

**Authors:** Addisie Geremew, Iris Stiers, Tim Sierens, Alemayehu Kefalew, Ludwig Triest

**Affiliations:** 1 Laboratory of Plant Biology and Nature Management (APNA), Department of Biology, Vrije Universiteit Brussel (VUB), Brussels, Belgium; 2 Department of Plant Biology and Biodiversity Management, College of Natural Sciences, Addis Ababa University, Addis Ababa, Ethiopia; Public Library of Science, UNITED KINGDOM

## Abstract

Land degradation and soil erosion in the upper catchments of tropical lakes fringed by papyrus vegetation can result in a sediment load gradient from land to lakeward. Understanding the dynamics of clonal modules (ramets and genets) and growth strategies of plants on such a gradient in both space and time is critical for exploring a species adaptation and processes regulating population structure and differentiation. We assessed the spatial and temporal dynamics in clonal growth, diversity, and structure of an emergent macrophyte, *Cyperus papyrus* (papyrus), in response to two contrasting sedimentation regimes by combining morphological traits and genotype data using 20 microsatellite markers. A total of 636 ramets from six permanent plots (18 x 30 m) in three Ethiopian papyrus swamps, each with discrete sedimentation regimes (high vs. low) were sampled for two years. We found that ramets under the high sedimentation regime (HSR) were significantly clumped and denser than the sparse and spreading ramets under the low sedimentation regime (LSR). The HSR resulted in significantly different ramets with short culm height and girth diameter as compared to the LSR. These results indicated that *C*. *papyrus* ameliorates the effect of sedimentation by shifting clonal growth strategy from guerrilla (in LSR) to phalanx (in HSR). Clonal richness, size, dominance, and clonal subrange differed significantly between sediment regimes and studied time periods. Each swamp under HSR revealed a significantly high clonal richness (*R* = 0.80) as compared to the LSR (*R* = 0.48). Such discrepancy in clonal richness reflected the occurrence of initial and repeated seedling recruitment strategies as a response to different sedimentation regimes. Overall, our spatial and short-term temporal observations highlighted that HSR enhances clonal richness and decreases clonal subrange owing to repeated seedling recruitment and genets turnover.

## Introduction

Clonality is a widespread life history trait in angiosperms that maximizes plant fitness [1, 2] and is significant for population persistence under a heterogeneous and stochastic environment [3, 4, 5]. However, the downside of persistent clonal reproduction is a complete loss of recombination that could lead to extinction [6]. Although the reaction norm is species dependent, clonal plants have developed different adaptive strategies to contrasting environmental gradients [5, 6,7]. Shifting clonal growth strategy is an adaptive mechanism crucial for colonization, establishment, and distribution of clonal plants [6, 8, 9, 10]. The two clonal growth strategies, guerrilla and phalanx, reveal endpoints of ramet arrangement in space [5, 11]. The guerrilla strategy produces loosely organized spreading growth modules that enable plants to escape adverse environmental conditions and competition [5, 11]. In contrast, the phalanx strategy results in clumped ramets occupying suitable patches to use profuse resources [5, 12]. Under heterogeneous environments, ramets expand and produce intermingled distribution among genets [13, 14]. Recent evidences show that the change in allele frequencies in response to an environmental gradient acts as an indicator of the effect of selective processes that modulate population genetic structure [15, 16]. Similarly, the shift in clonal growth strategy and genet frequencies may reflect how clonal plants are resilient to dynamic environmental factors that finally lead to clonal structuring and genetic divergence. The dynamics of the ramets and genets of clonal plants in both space and time is critical to explore species adaptation and processes regulating population differentiation [14]. In addition to the clonal growth strategy, clonal plants have the capacity to reproduce both sexually and vegetatively, and the balance between these reproduction modes regulates how genets colonize space and recruitment [17]. Depending on the turnover of genets, two recruitment strategies have been known for clonal plants [18]: (i) the ‘Initial Seedling Recruitment’ (ISR) strategy; and (ii) the ‘Repeated Seedling Recruitment’ (RSR) strategy. Under the ISR strategy, the development of a population includes selective elimination of genotypes, as a result the population ends up with a small number of large and evenly aged clones. In contrast, RSR strategy enables survival of small clones of different age and size to coexist and leads to high genetic and clonal diversity within a population [19]. Differential intensity between these recruitment strategies dictates clonal richness [19, 20] and contributes to patch growth, clone coexistence and evolution, and disturbance recovery [18, 21]. Therefore, in examining the populations of a species occurring in different environments, it is important to explore how each reproduction mode and recruitment strategy contributes to genets distribution and dynamic.

Sedimentation affects plant growth and distribution in lacustrine wetlands by altering moisture level, aeration, temperature and other factors of substrate-plant-microenvironment conditions [22, 23]. Despite contradictory conclusions, a shift in clonal growth strategy (from phalanx to guerrilla and vice versa) has been reported as an adaptive mechanism to sediment burial by a few wetland and costal dune plants such as *Suaeda salsa* [24], *Phalaris arundinacea* [25] and *Carex brevicuspis* [11]. Arnaud-Haond et al. [26] found no significant correlation between sedimentation rate and plasticity of clonal traits in a seagrass but rather, with the demographic parameter, mortality. More specifically, severe land degradation and soil erosion in the upper catchment of tropical lakes bordered by *Cyperus papyrus* L. dominated vegetation result in a sediment load gradient from land to lake ward [27]. Investigating the shift in clonal growth strategy, exploring the genotypic diversity and structure metrics along such a sediment gradient may shed light on the resilience of *C*. *papyrus* to sediment accretion and help to forecast the evolutionary potential of a population [28]. A high level of genotypic diversity is related to increased resistance [29] and resilience [30]. It is possible that the spatial variability of sediment loads can create microenvironments that are significant for the persistence of multiple genotypes through seedling recruitment, and at the same time favor directional selection and increase genotypic diversity within a population [31].

Biologically productive papyrus swamps, dominated by the keystone species paper reed (*Cyperus papyrus* L) have been widely recognized for their valuable regulatory and provisional ecosystem services [32, 33, 34, 35, 36, 37, 38, 39]. More explicitly, these swamps control sediment load into the adjoining waterbodies [40, 41, 42]. Recent quantitative studies have shown that catchments with disturbed papyrus swamps resulted in sediment yield three times greater than those with pristine papyrus vegetation [43, 44]. Since most of the papyrus swamps are threatened by anthropogenic pressures [27, 37], there is an imperative need for restoration and management measures to reduce sediment accretion in adjacent lakes and wetlands, supported by serious study of the population dynamics and adaptive response of the dominant and keystone species, *C*. *papyrus*. The effect of sedimentation on the growth, distribution and diversity of clonal plants in coastal ecosystems is well known [7, 45, 46]. In contrast, to our knowledge, there is less information on the effect of sedimentation on the responsive survival strategy, i.e. dynamics in seedling recruitment strategy, clonal growth strategy, structure and diversity of emergent macrophytes at the temporal and spatial scales.

In this study, we combined morphological traits and genotype data using 20 novel microsatellite markers to assess the spatial and temporal dynamics in clonal growth, diversity and structure of the paper reed (*C*. *papyrus*), as a response to sedimentation regimes. We hypothesized that a higher level of sedimentation would lead to: (1) increased occurrence of phalanx strategy over guerrilla strategy; (2) increased ramet density and genet size; and (3) higher genotypic diversity through seedling recruitment. Our findings contribute to the understanding of population dynamics, resilience and survival strategy of clonal plants, especially of the keystone species *C*. *papyrus* in the context of sedimentation. It will also provide an important guideline for designing conservation and restoration plans of *C*. *papyrus* and protection of lakes from over-sedimentation.

## Materials and methods

### Species description

The paper reed, *Cyperus papyrus* (Cyperaceae), is a perennial rhizomatous keystone emergent species native to Africa that occurs particularly along freshwater lakes, rivers and stream margins. In the Lake Tana region, papyrus forms monocultures and co-occurs with other emergent macrophytes, such as *Typha latifolia*, *Polygonum senegalense*, *Phragmites australis*, *Vossia cuspidata*, *Echinochloa stagnina* and *E*. *pyramidalis* [47]. The species occupied the center of the lake and its basin between 16,700 and 15,100 BP following an intermittent flooding event after draw down [48]. The species produces inter-meshed horizontal rhizomes that shield the lake and adjacent wetlands from over-sedimentation and pollutants [40]. Reproduction is primarily sexual via very small seeds and asexual through extension of horizontally creeping rhizomes. In the Lake Tana basin, it flowers and fruits from June to August during the rainy season. Due to over-exploitation and ongoing habitat conversion [27], *C*. *papyru*s is locally on the brink of extinction. The short term threats come from direct habitat loss and damage from over-collection [49].

### Study area and sampling sites

**Fig 1:**
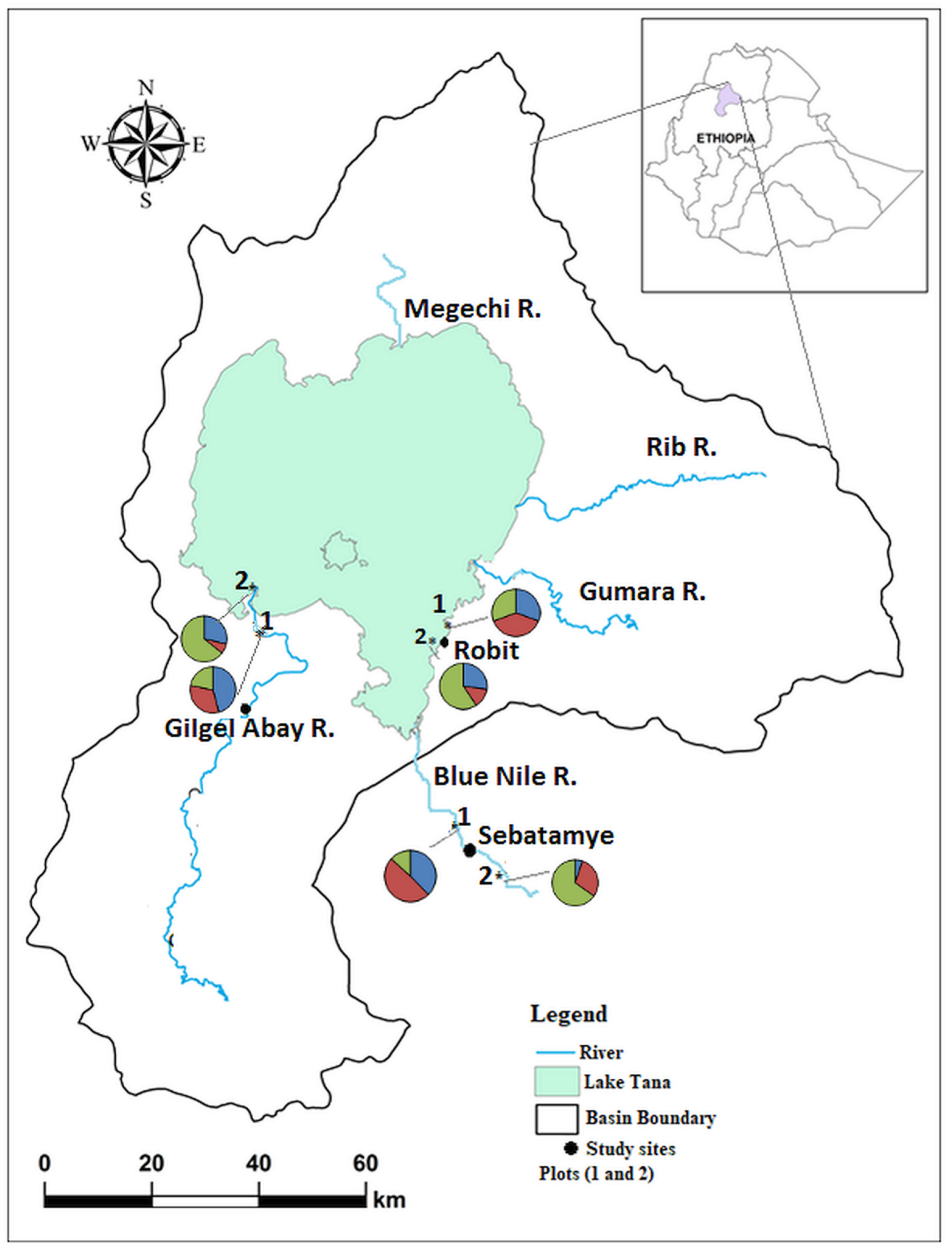
Map of Lake Tana with the papyrus swamps studied and the proportion of genets evolved temporally across two sediment regimes. Blue represents genets only evolved in 2014, red only in 2016, and green in both years. Where 1 = HSR and 2 = LSR The map is reprinted from Chebud and Melese [57] under a CC BY license, with permission from [John Wiley and Sons], and with the original copyright [2009] used as a shapefile.

The field study was carried out in three papyrus wetlands situated in the Lake Tana basin (Fig 1), which is perched within a large dome of the Ethiopian highland. This large freshwater body (area 3,200 km^2^; mean depth 9 m) along the Blue Nile River is adjoined by seasonally and permanently flooded wetlands and also fed by a dendritic network of rivers [50]. The climate in the basin is a tropical monsoon with a seasonal distribution of precipitation gauged by a shift in the inter-tropical convergence zone. Comprehensive accounts referred to the hydrology, climate, and lake level fluctuations [43, 51, 52]. The islands, peninsulas, adjacent wetlands, and flat depression areas are characterized by several soil types (e.g., Vertisols, Chromic Luvisols, Eutric Luvisols, and Lithic Leptosols). Because of severe land degradation and soil erosion in the adjoining landscape [53], alluvial sand deposition around the lake is amplified [43, 44, 54].

Three papyrus swamps, namely Gilgel Abay, Robit, and Sebatamye, were selected by considering differences in depth, large coefficients of runoff, and sediment deposition gradients [55] from the shore of the lake (Fig 1; Table 2). The Robit swamp has inundation-drawdown cycles and agricultural encroachment in the surrounding landscape. Three perennial rivers from the east Tana catchment, the Gumara, Rib, and Gelda deposit a large proportion of the sediment load to Robit and other adjacent swamps resulting in sharp sedimentation gradients. The papyrus swamp located on the Gilgel Abay represents the stretches of the floodplain in the upper Blue Nile River in west Tana. The Gilgel Abay river delivers the highest amount (29%) of sediment load into Lake Tana [56] and forms a visible delta with a clear gradient. The upper catchment of this area is also heavily disturbed and characterized by little vegetation cover. Unlike the Gilgel Abay and Robit swamps, the Sebatamye swamp is characterized by sediment depositions that were exported out of the lake via the Blue Nile River. This swamp is also typified by steep slopes and rocky substratum ahead of alluvial sediment deposition along the bank of the Blue Nile River and is upstream from the 40m Tis Isat fall.

### Sampling design and sample collection

The field study was performed in 2014 and 2016. In 2013, a preliminary survey on the sediment gradient and vegetation zonation was conducted at the beginning of the rainy season. To verify sedimentation level and its effect on the depth of the wetland and the lake, wooden sticks of 10 m long were placed randomly until the next dry season. At each swamp, a transect was established landward to lakeward along the sediment gradients. Two rectangular plots, each 18 m x 30 m separated by 500 m, were established on two distinct sediment regimes: a high sediment level corresponded to the most shallow section (0.9 m sediment level) and a very low sediment regime represented a relatively deeper section of the lake (< 0.2 m sediment level). To characterize the clonal traits of papyrus, such as girth diameter, culm height, ramet density, spacer length (i.e. space between individual stands (shoots)), and biomass, ten 3 m x 3 m quadrats were delineated with cords in each plot. We used a Vernier caliper to measure girth diameter. Ramet density in a plot was estimated as the mean of the number of shoots per quadrat. Above the substrate, papyrus plants (n = 9 per quadrat) were clipped and sorted into leaves and culm. Subsequently, these parts were oven-dried at 70°C for 48 hours and weighed. Absolute aboveground biomass was determined as the sum of dried weights of leaves and culm. To monitor the temporal dynamics of ramets and genets and to avoid resampling (DNA extractions) of the same genet in 2016, all aerial shoots observed in 2014 within each plot were tagged using small, colored plastic strings.

To examine the pattern and dynamics of clonal diversity and structure, a total of 322 and 314 ramets shoots (40 to 60 per plot) were collected in 2014 and 2016, respectively. In order to minimize sampling of duplicates, individuals were sampled at an interval of three meters. The location of each sampling plot was recorded using a handheld GPS (Garmin 45XL, USA). Samples were dried with silica gel pending DNA extraction.

### DNA extraction and microsatellite generation

Since our preliminary test using 11 microsatellite primers [58] showed low polymorphism in *C*. *papyrus* populations of the Ethiopian highlands, additional polymorphic markers were required for an in-depth study of population connectivity, clonality, and seedling recruitment. To do so, genomic DNA was extracted from lyophilized green umbel rays (20 mg) of *C*. *papyrus* collected from Lake Tana (Ethiopia, 11°37.336’ N, 37°20.542’) using the E.Z.N.A. SP plant DNA Mini Kit (Omega Bio-tek). The quantity of the DNA was determined by NanoDrop One Spectrophotometer (Thermo Scientific). Also, the quality of the DNA was evaluated following library DNA QC criteria. Twenty-five ng of DNA was subjected to Illumina sequencing on a HiSeq, 100 bp paired-end library (Macrogen, Seoul, Republic of Korea). A total of 43.83 million reads of at least 80 bp, without a tag and adapter, were generated in FASTQ format, and we converted the FASTQ files into FASTA files using NET Bio.1.1 (https://bio.codeplex.com). Both unassembled reads and contigs were applied to identify microsatellites and design primers using QDD2 v.2 [59] integrated with Primer3 [60]. After discarding the loci that showed compound SSR stretches, 5,510 target microsatellites displayed at least five pure repeats, a size of 100 bp PCR product and lowest penalty values. Subsequently, 70 primer pairs were selected for synthesis (Sigma Aldrich, Germany) and tested for amplification on eight papyrus samples.

### Primer testing and multiplexing

Simplex PCRs were conducted in a total volume of 20 μL per reaction and contained: 0.2 mM of each dNTP (Promega), 2 mM MgCl_2,_ 0.5 μM of each forward and reverse primer (Sigma), 0.1 mM BSA, 1 U *Taq* DNA polymerase (Promega), and 3 μl (25 ng) template DNA. The PCRs were carried on a Bio-Rad Thermal Cycler using the profile of initial denaturation at 95°C for 3 min, followed by 35 cycles of 95°C for 0.4 min, annealing at 54°C for 1 min, extension at 72°C for 2 min and a final extension at 72°C for 5 min. To separate the PCR products we used capillary electrophoresis (QIAxcel^®^ Advanced System). The QX alignment marker of 15 bp/1600 bp (1.5 μl) and the QX size marker of 25 bp to 500 bp (5 μl) were employed. Fragments or allele size were visualized and established with QIAxcel^®^ Screen gel software that provides both electropherogram and gel images of DNA separation. Out of the 70 microsatellite loci tested, 30 produced scorable amplified products. A final subset of nine novel loci that appeared polymorphic and heterozygous, were fluorescently labeled (forward primers, Life Technologies, USA) and multiplexed in two sets, including the eleven primers developed by Triest et al. [58] using Multiplex Manager [61].

Multiplex PCRs were carried out for 420 samples from the three swamps within the Lake Tana basin in a 12.5 μL mix containing 6.25 μL 1x QIAGEN Master Mix (Valencia, USA), 2.5 μL of RNase free water, 2.5 μL of 25 ng DNA, and 1.25 μL of primers mix (see Triest et al.[58] for the reaction profile). The PCR products were resolved using ABI 3730xl Genetic Analyzer and fragment sizes were determined with an internal size standard GeneScan500 LIZ in Macrogen (Seoul, Republic of Korea). Allele sizes for each microsatellite locus per individual were scored with GeneMarker v2.4.1 (SoftGenetics, USA).

### Data analysis

GenAlEx v. 6.5 [62] was employed to compute the number of alleles (N_A_), expected (*H*_E_) and observed (*H*_O_) heterozygosity, and to assess departure from the Hardy–Weinberg equilibrium (HWE) at locus level. We also analyzed inbreeding coefficient (F_IS_) and linkage disequilibrium (LD) among the 20 microsatellite loci implemented in FSTAT version 2.9.3.2 [63]. The test for presence of null alleles, large allele dropout, and genotyping errors was done with MICRO-CHECKER version 2.2.3 [64].

Clonal diversity was measured by different parameters using GENCLONE 2.0 [65]. Clonal richness (*R*) was estimated from the number of distinct multilocus genotypes (MLGs) (*G*) and the number of ramets (N) [66]. To estimate a comparable *R* across populations, we standardized the number of sample units collected (N) by the proportion of sample units collected at the site with higher density (D_max_):

$$Ns=\frac{N}{D\text{max}}$$ (1)

We also applied the Pareto distribution index (β) as a proxy for clonal diversity [67] and the dispersion of ramets among genets. Spatial clone architecture and clonal structure were estimated among the six sampling plots (two plots per site) in three swamps by calculating the aggregation index (*Ac*) at plot level and clonal dominance index (*Dc*) for each genet using GENCLONE under 10, 000 permutations. *Ac* was computed as:

$$Ac=\frac{\left(Psg-Psp\right)}{Psp}$$ (2)

where *P*_*sg*_ is the mean likelihood of clonal identity of all sample unit pairs, and *P*_*sp*_ is the average probability of clonal identity among pairwise nearest neighbors. To determine the degree of intermingling among genets, *Dc* was computed for each genet as:

$$Dc=\frac{\left(NR-1\right)}{\left(NT-1\right)}$$ (3)

where *NR* is the genet size (i.e. genet size is the number of ramets per genet) and *NT* is the total number of ramets sampled within the genet range[68]. A *Dc* index of one points out that the spatial range of a genet is occupied fully by ramets of the identical genet. Only genets signifying more than two ramets were accounted for the calculation of *Dc*. Despite the edge effect was minimized by sampling across the center of a plot, we tested for its effect on dispersion of distinct or rare MLGs that overvalue clonal diversity following Arnaud-Haond et al. [67]. To estimate the minimum spatial extent of the largest clone in each plot, we determined the clonal subrange (CR) which corresponds to the maximum distance between two identical MLGs belonging to the same clone in meters. In other words, it is measured as the distance for which the probability of clonal identity becomes null [69]. Temporal and spatial differences in clonal growth, genotypic diversity, and structure measures were analyzed with a non-parametric Mann–Whitney U-test using SPSS XII (SPSS, Chicago). Spearman’s correlation coefficients were used to examine relationships between estimates of genotypic diversity, structure, and clonal growth traits. Furthermore a linear regression was carried out to assess the relationship between spacer length and ramet density.

## Results

### Characteristics of the isolated microsatellites

The 20 microsatellite loci were highly variable, having 3 (Cypap 62) to 12 (Cypap 23) alleles per locus over 282 ramets yielding 141 individuals (three populations) with distinct multilocus genotypes (MLGs) (Table 1). Levels of expected and observed heterozygosities per locus varied from 0.41 to 0.86 and from 0.25 to 0.87, respectively (Table 1). There was no evidence for large allele dropout or genotyping error. Putative null alleles were detected at only one locus (Cypap 28) in one population, with a very low frequency because of excess homozygotes (P < 0.05). The microsatellite loci developed and multiplexed showed a wide range of inbreeding coefficient (*F*_*IS*_) of -0.046 (Cypap 10) to 0.611 (Cypap 15). The within-population tests revealed that there was no significant departure from the HWE (P < 0.001) nor LD between loci in the populations and thus, further analyses of clonal diversity and structures were carried out using these 20 loci.

**Table 1:** Genetic diversity measures and characteristics of twenty microsatellite loci from *C*. *papyrus* genets (Total N = 141, per population = 47) of three populations of Lake Tana (Ethiopia).

Loci	Primer sequences (5’-3’)	Repeat motif	Accession No.	*T*_*an*_	Allele size	*A*_*T*_	*A*_*E*_	*H*_*O*_	*H*_*E*_	*F*_*IS*_^*(HWE)*^
Cypap22^++^	F: PET-TGGAACTTACAAGCCATACAGATTC R: CACGGTCAAATGTCTACCAGC	(AAG)14	KT873448	57	90–115	7	4.7	0.504	0.439	-0.148^ns^
Cypap23^++^	F: NED-TGTCCTAATGTTGTTGAATGCTT R: TTGAACAGATTGGAAGTTTCTTT	(AG)9	KT873449	57	98–135	12	7.6	0.871	0.591	-0.476^ns^
Cypap27^++^	F: VIC-CATGGCTCCCGTGTTAACTT R: CAAGTATGACTCCAAGCATTTCT	(AT)8	KT873450	57	82–124	6	4.3	0.393	0.762	0.491^ns^
Cypap28^++^	F: 6FAM-ACTCACCCACACAGTCACACT R:TACCAGTGTCGCATCTGCAT	(ACG)9	KT873451	57	100–120	6	4.6	0.431	0.782	0.478**^null^
Cypap34^+^	F: VIC-TCATATCACTATATCAGTCTATCAGGG R: GACACAGGCACACCCAGAA	(AAAGG)8	KT873452	57	90–118	10	6.3	0.508	0.834	0.446^ns^
Cypap38^+^	F: 6FAM-AAGGTAATCAATCTGGTCTGCTG R: CCACTTCTCTTTCTCCTCTCTCAA	(AG)11	KT873453	57	90–112	9	6.6	0.474	0.447	-0.062^ns^
Cypap52^+^	F: 6FAM-CCAAACCCCAACAGAGCAAA R: ACTTCGGGTGGGATCAAACT	(CCA)9	KT873455	57	218–246	8	5.9	0.419	0.820	0.594^ns^
Cypap56^++^	F: 6FAM-GGGGACAATGGCAAAGCTAC R: TGAACTCTGAAAGACTGAAACCA	(GA)15	KT873456	57	230–260	6	4.6	0.326	0.768	0.592^ns^
Cypap62^++^	F: PET-GAGAGAGGCACCTGACCTAGC R: TGGTATCATTGTCCATGTTTGC	(AG)5	KT873454	57	130–140	3	2.4	0.247	0.552	0.573^ns^
Cypap4^+^	F:6FAM-AACAAGTTCATTAGTCATGGAGTG R: TGTTCTCTTGTGGCTCCTGA	(TG) 10	KC460659	57	138–169	10	7.0	0.494	0.856	0.371^ns^
Cypap14^+^	F:6FAM-CATGCACATGCTTTTGATGA R: TGTTCATTGATCGTGCCTTT	(GT)n	KC460665	57	186–200	5	4.5	0.384	0.776	0.559^ns^
Cypap10^+^	F: VIC-GACAGCGGCTTGTTTTAAGG R: TCTCTGCCTTTCACACACTCA	(GT)7	KC460662	57	143–159	5	3.3	0.565	0.529	-0.046^ns^
Cypap13F^+^	F: NED-CTGTGGCATGGCATCAAAT R: AAGCACAGGGGTTATGGTTG	(GT)9(AAT)4	KC460664	57	151–160	4	2.4	0.290	0.575	0.393^ns^
Cypap13S^+^	F: NED-CTGTGGCATGGCATCAAAT R: AAGCACAGGGGTTATGGTTG	(GT)9 (AAT)4	KC460664	57	163–175	4	3.4	0.409	0.699	0.258 ^ns^
Cypap5^+^	F: NED-TGAGTTAATTGGGCCTCCAC R: ATCTGACGCGACTTGTTCCT	(CT)13	KC460660	57	219–248	7	3.1	0.326	0.666	0.583^ns^
Cypap3^+^	F: PET-AAAAGGATTCGATCTGTCACG R: AAGGGGAAACTTGGTCCTGT	(CT)14(GTGTAA)2	KC460658	57	154–217	5	3.3	0.408	0.684	0.561^ns^
Cypap7^++^	F: 6FAM-GAAGCCAGAGGGAAAGTGTG R: CAAAGCAAACCAGCTCCTGT	(GT)6GC(GT)6(GA)9	KC460661	57	172–188	7	4.6	0.429	0.783	0.453^ns^
Cypap12^++^	F: VIC-TGATTTCCTCGCAGCCTAGA R:AGACCCACAACCCACAAAAA	(TC)8	KC460663	57	176–182	5	4.1	0.418	0.750	0.452^ns^
Cypap15^++^	F: NED-CGGAGAACATGTCCTAAATGC R: GGAAAGCAGAGAGCATAGCC	(CT)7(CA)6	KC460666	57	164–182	6	3.7	0.409	0.718	0.611^ns^
Cypap1^++^	F: PET-AAGCAGCAAATGAGACAACAA R: TGTTGGTTGGTTGGTGAAAA	(CAGA)10	KC460657	57	173–217	11	7.3	0.466	0.857	0.583^ns^
Mean	_	_	_	_	_	6.8	4.7	0.438	0.694	0.335

*Tan* annealing temperature, *A*_*T*_ total number of alleles, *A*_*E*_ effective number of alleles, *H*_*O*_ observed heterozygosity, *H*_*E*_ expected heterozygosity, *F*_*IS*_ inbreeding coefficient with associated tests of deviation from Hardy-Weinberg equilibrium (HWE, ns = not significant,

** significant at 0.01 level), F hex-labelled forward primer, R reverse primer,

^+^ locus in multiplex-1,

^++^ locus in multiplex-2,

^null^ null alleles, shaded primers developed by Triest et al. [58] and non shaded, new primers.

### Clonal growth metrics

All clonal growth metrics (culm height, ramet density, girth diameter, spacer length, and biomass) of *C*. *papyrus* were significantly different between the high sedimentation regime (HSR) and low sedimentation regimes (LSR) (Table 2). Mean culm height and girth diameter were about twice higher in the LSR compared with the HSR which contained dense ramets with short culms. Ramet density also varied by almost three orders of magnitude (Table 2), between the sedimentation regimes. The maximum density of *C*. *papyrus* ramets was 57.6m ^-2^ at Robit swamp. Clumped ramets of HSR populations produced shorter spacer lengths over spreading ramets than did those in the LSR (Table 2), reflecting HSR favors phalanx strategy than the LSR that promotes the guerrilla strategy. Papyrus under an HSR demonstrated significant temporal difference in ramet density and spacer length (S1 Fig).

**Table 2:** Clonal growth traits of *C*. *papyrus* across sedimentation regimes.

Swamp	Plot	Latitude (N)	Longitude (E)	Altitude (m)	Sediment regime	Ramet density (m^-2^)	Culm height (cm)	Girth diameter (cm)	Spacer length (cm)	Biomass (gm^-2^)
Robit	1	11°44’24.51”	37°25’22.42”	1788	High	57.6 (1.85)	307.9 (11.2)	7.4 (0.54)	48.4 (2.66)	5749.9 (93.1)
	2	11°44’16.13”	37°25’18.00”	1788	Low	17.0 (1.19)	463.5 (10.9)	16.2 (1.04)	64.3 (1.75)	4609.1 (96.1)
Sebatamye	1	11°30’57.02”	37°30’36.22”	1691	High	41.0 (1.93)	240.7 (14.5)	6.8 (0.34)	55.5 (1.79)	5127.6 (108)
	2	11°30’57.63”	37°30’35.54”	1690	Low	11.8 (1.02)	366.5 (5.03)	13.6 (1.01)	73.7 (1.31)	3491.0 (71.6)
Gilgel Abay	1	11°52’00.22”	37°06’51.82”	1788	High	44.0 (2.75)	326.2 (7.84)	5.3 (0.39)	53.0 (1.98)	9073.5 (241)
	2	11°51’43.88”	37°06’41.17”	1788	Low	15.0 (1.02)	381.3 (6.73)	15.3 (15.5)	66.6 (1.52)	4199.6 (75.6)
Mean HSR		_	_	_	_	47.5^a^	291.6^a^	6.5^a^	52.3^a^	6650.3^a^
Mean LSR		_	_	_	_	14.6^b^	403.8^b^	15.0^b^	68.2^b^	4099.9^b^

The significance level is based on T-test for two samples, d.f = 1 and P < 0.05). Different letters indicate significant difference between sedimentation regimes. Values in the bracket show standard error of the mean based on n = 50 (five plants per quadrat for total of 10 quadrats per swamp).

### Dynamics of ramets and genotypes composition

**Fig 2:**
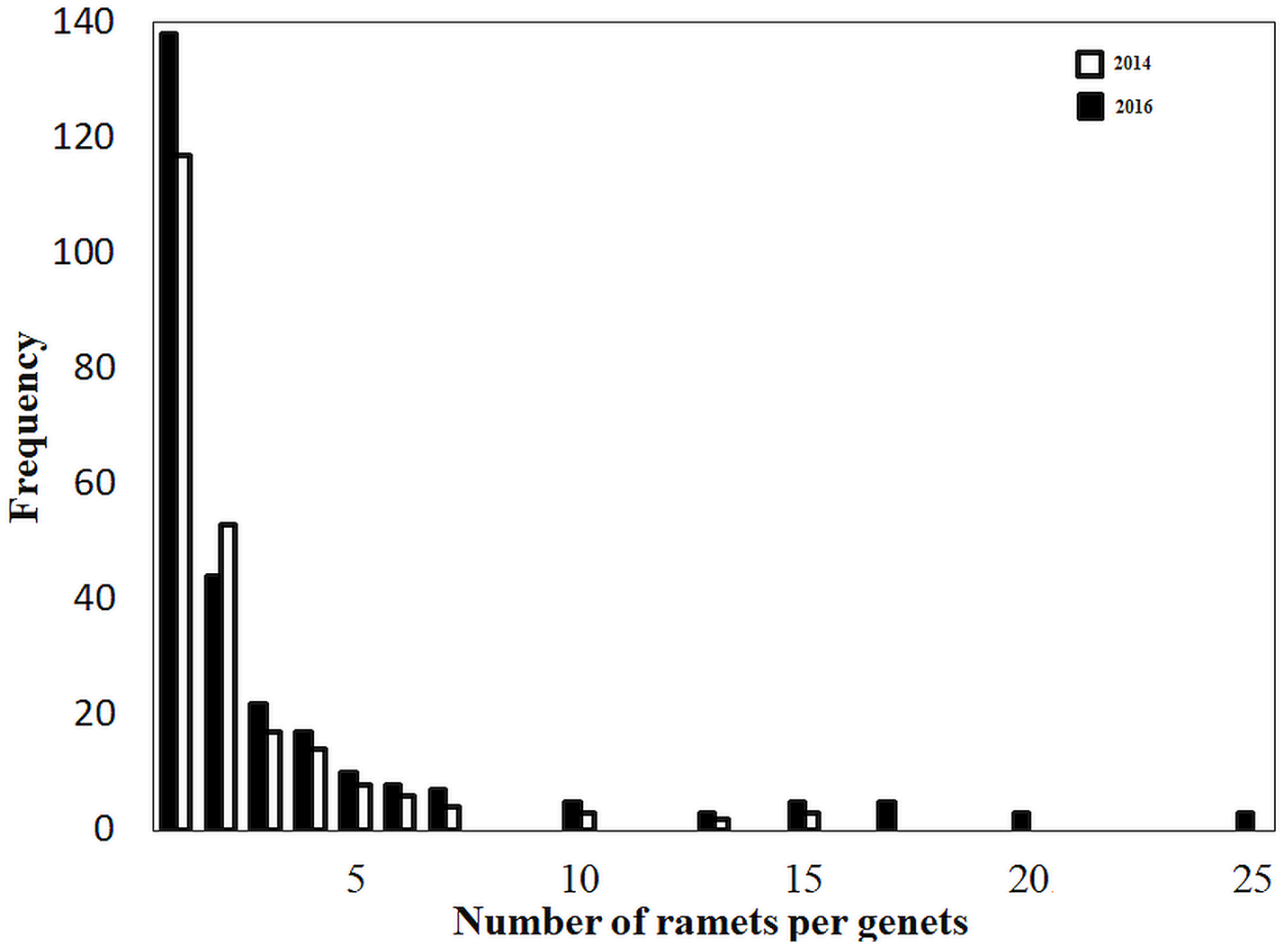
Frequency distribution of genets (genet size) for populations of *C*. *papyrus* across papyrus swamps in Lake Tana basin, Ethiopia for 2014 and 2016 pooled over two contrasting sedimentation regimes.

Permutation tests for genotypic resolution revealed an exponential increase with the number of loci and then an asymptotic trend subsequent to the addition of locus (S2 Fig). We identified a total of 408 distinct MLGs across the 636 ramets (N = 301 for 2014 and N = 335 for 2016). The likelihood of encountering identical MLGs twice because of random sexual recombination events was very low (*P*_sex_ < 0.01, Table 3). Frequency distribution of allelic distances between MLGs confirmed that MLGs diverged neither as a result of scoring error nor from somatic mutation (S3 Fig). The number of individuals with distinct MLGs across sedimentation regimes ranged widely among the swamps studied (HSR: 39–53; LSR: 19–39; Table 3) and differed significantly between years (Mann–Whitney test: U = 55.5; P < 0.05). In general, clone composition differed between sedimentation regimes and demonstrated temporal dynamics within each papyrus swamp (Fig 1). Ramets belonging to each genet were distributed in a leptokurtic pattern (Fig 2). The majority of clones (particularly in LSR) were designated by more than two *C*. *papyrus* ramets, and eight clones were found in more than one sedimentation regime and swamp.

**Table 3:** Clonal diversity and structure measures of *C*. *papyrus* ramets sampled in three papyrus swamps in Lake Tana under two sedimentation regimes during 2014 and 2016.

Swamp	Plot	Sediment regime	Year	Clonal diversity descriptors							Clonal structure				
				N	G	*P*_*sex*. *max*_	*R*	*D*	*E*	*β*	*NR*	*DC*	*Ac*	*E*_*e*_	*CR*
Robit	1	High	2014	60	48	9.11 x 10−^19^	0.79	0.99	0.92	3.58	12	0.186	0.194*	0.031^ns^	11
			2016	60	52	4.13 x 10^−15^	0.86	0.99	0.79	2.95	8	0.119	0.146*	0.023^ns^	9.2
	2	Low	2014	60	22	6.42 x 10^−6^	0.36	0.97	0.88	2.08	38	0.627	0.319*	0.043^ns^	25
			2016	39	19	6.86x 10^−17^	0.47	0.96	0.83	2.04	20	0.500	0.385*	0.110^ns^	27
Sebatamye	1	High	2014	60	39	4.12 x 10^−16^	0.64	0.98	0.96	2.61	21	0.339	0.358*	0.141	10.1
			2016	60	53	1.64 x 10^−20^	0.88	0.97	0.79	3.12	7	0.102	0.129*	0.061^ns^	9.1
	2	Low	2014	41	21	4.75 x 10^−17^	0.50	0.99	0.82	ǂ	20	0.475	0.141*	0.015^ns^	18
			2016	50	24	1.5 x 10^−7^	0.47	0.96	0.7	1.77	36	0.714	0.209*	0.014^ns^	37
Gilgel Abay	1	High	2014	60	35	9.8 x 10−^17^	0.58	0.98	0.96	2.87	25	0.407	0.390*	0.289^ns^	14.6
			2016	60	47	3.03 x 10^−20^	0.78	0.99	0.94	3.317	13	0.203	0.277*	0.039^ns^	10.4
	2	Low	2014	41	28	4.6 x 10−^17^	0.67	0.98	0.92	2.32	13	0.300	0.304*	0.161^ns^	17
			2016	45	20	5.13 x 10^−9^	0.43	0.99	0.91	3.33	25	0.545	0.101*	0.053^ns^	22
Mean HSR				60	45.7^a^	_	0.76^a^	0.98^a^	0.89^a^	3.07^a^	14.3^a^	0.226^a^	0.249^a^	0.01^a^	10.6^a^
Mean LSR				46	22.3^b^	_	0.48^b^	0.97^a^	0.84^a^	2.31^b^	25.3^b^	0.527^b^	0.243^a^	0.07^a^	24.3^b^

N: number of ramets, G: number of MLGs, NR: clone size (m^2^), R: clonal richness. D and E: Simpson index and its equitability index. *β*: slope of Pareto distribution, D_C_: clonal dominance, A_C_: aggregation index, E_e_: edge effect,

^ǂ^:multilocus genotypes reached maximum and CR: clonal subrange. All measures of clonal structure were obtained after 1000 permutations, ns, not significant and

*P < 0.05. Different letters indicate significant difference between sedimentation regimes collated for the three swamps (P < 0.05).

### Clonal diversity metrics

Clonal richness (*R*), after being standardized for different sample sizes, ranged from 0.36 (Robit, 2014) to 0.67 (Gilgel Abay, 2014) and from 0.58 (Gilgel Abay, 2014) to 0.88 (Sebatamye, 2016) for LSR and HSR, respectively (Table 3). On the overall data set, mean *R* of 2014 and 2016 were significantly different (Mann–Whitney test: U = 45.3; P < 0.05). We also found *R* to be significantly different between sedimentation regimes (Mann–Whitney test: U = 65; P < 0.05). Simpson’s diversity (*D*) and evenness (*E*) indices were steady when compared across swamps and sampling periods (Table 3). The Pareto descriptor (β) of clonal diversity estimate showed significant spatial heterogeneity of genets distribution resulting different clone sizes in response to sedimentation (HSR: mean β = 3.07; LSR: mean β = 2.31). Similarly, Pareto distribution showed a temporal variability (mean 2014 β = 2.19 and mean 2016 β = 2.75). The lowest β was observed in the Sebatamye swamp plot 2 (β = 1.77) in 2016.

### Clonal structure changes in space and time

Clonal dominance (*Dc*) spanned from 0.10 (Sebatamye 2016, HSR) to 0.5 (Robit 2016, LSR) (Table 3), indicating that the majority of clones were intermingled. The difference in mean *Dc* in the two sedimentation regimes was significant (Mann–Whitney test: U = 52; P < 0.05). Clone size was unevenly distributed among genotypes and the largest clone size (*NR* = 38) was detected in Robit swamp under LSR in 2014 (Table 3). With the presumption that genotypes are randomly distributed in space, permutation analysis revealed significant aggregation of ramets within genets of each papyrus swamp plot (range *Ac* = 0.1–0.4). However, the mean *Ac* was neither significantly different between sedimentation regimes (Mann–Whitney test: U = 44.1; P > 0.05; Table 3) nor between years (Mann–Whitney test: U = 47; P > 0.05). The test for the maximum spatial linear limits (clonal subrange, CR), where the chance of two identical MLGs of the same clone becomes null, varied from 9.1 m to 37 m. Plots with a high number of distinct genotypes and clonal richness were found to have a minimum clonal subrange (Table 3). There was no evidence of evidence of a significant edge effect detected within plots in either sedimentation regime or sampling year, except in Sebatamye during 2014. The edge effect did not have a sheer influence on the genotype distribution as observed.

### Association between clonal growth traits and diversity

**Fig 3:**
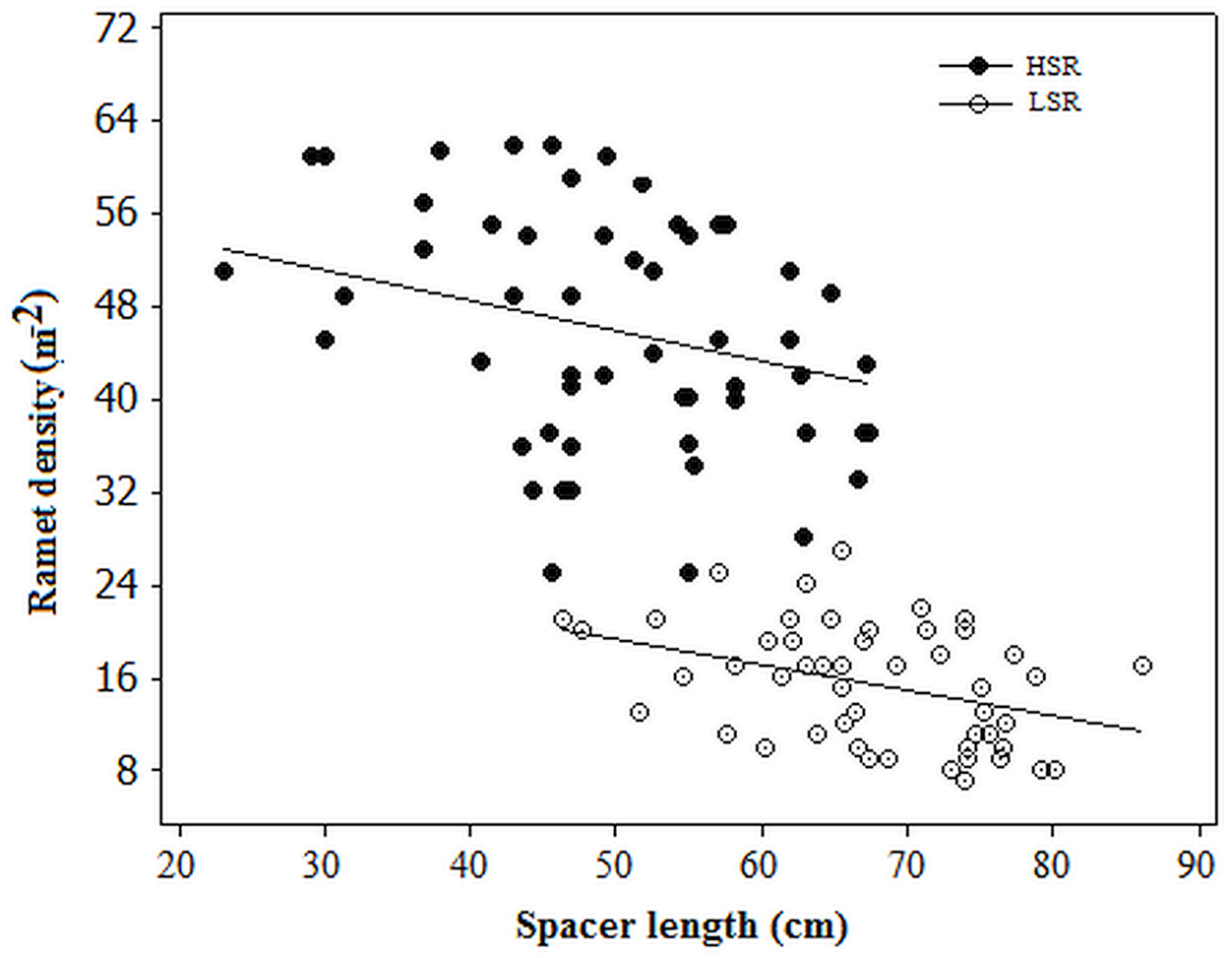
Relationship between spacer length and ramet density of *C*. *papyrus* populations in two sedimentation regimes pooled over 2014 and 2016 (n = 18 per quadrates of each plots). R^2^ = 57.7; P < 0.05 for LSR and R^2^ = 42; P < 0.05 for HSR.

We tested the relationship of clonal growth traits with clonal structure and with diversity (Table 4). Ramet density corresponded to a decrease in girth diameter (r = -0.60; P < 0.05), spacer length (r = -1.00; P < 0.01; Fig 3), culm height (r = -0.60; P < 0.05), clonal dominance (r = -0.71; P < 0.05) and clonal subrange (r = -0.72; P < 0.01) but to an increase in clonal richness (r = 0.65; P < 0.05) and aboveground biomass (r = 0.94; P < 0.01). Mean clone size was significantly negatively correlated with clonal richness (r = -0.91; P < 0.01; Table 4) and number of distinct genotypes (r = -0.68; P < 0.05) and positively with *Dc* (r = 0.92; P < 0.01), indicating that with an increase in of few, large dominant clones, there is a decrease in clonal richness.

**Table 4:** Relationship between clonal growth, diversity and structure parameters for papyrus populations data collated for two sediment regimes in two years (Spearman’s correlation coefficient).

	**Ramet density**	**Girth diameter**	**Culm height**	**Spacer length**	**Biomass**	***G***	***R***	***D***	***E***	***β***	***NR***	***DC***	***AC***	***CR***
**Ramet density**														
**Girth diameter**	-0.600*													
**Culm height**	-0.600*	0.829**												
**Spacer length**	-1.000**	0.600*	0.600*											
**Biomass**	0.943**	-0.714**	-0.543	-0.943**										
***G***	0.735**	-0.735**	-0.862**	-0.735**	0.664*									
***R***	0.651*	-0.651*	-0.793**	-0.651*	0.566	0.904**								
***D***	0.348	-0.326	-0.289	-0.348	0.289	0.249	0.331							
***E***	0.362	-0.384	-0.128	-0.362	0.512	0.063	0.028	0.3						
***β***	0.678*	-0.452	-0.466	-0.678*	0.636*	0.559	0.501	0.543	0.362					
***NR***	-0.512	0.355	0.554	0.512	-0.384	-0.682*	-0.907**	-0.356	0.131	-0.446				
***DC***	-0.707*	0.594*	0.735**	0.707*	-0.622*	-0.860**	-0.970**	-0.407	-0.12	-0.580*	0.924**			
***AC***	0.155	-0.028	0.212	-0.155	0.297	-0.182	-0.242	-0.462	0.531	-0.336	0.38	0.238		
***CR***	-0.722**	0.729**	0.857**	0.722**	-0.673*	-0.879**	-0.877**	-0.45	-0.23	-0.578*	0.732**	0.914**	0.203	

** significant at 0.01 level and

* significant at 0.05 levels

## Discussion

In this study, combining clonal traits and genetic data, we found a trade-off between phalanx strategy and guerrilla strategy under contrasting sedimentation regimes. Higher levels of sedimentation led to an increase in ramet density, enhanced phalanx strategy and favored higher clonal diversity which can be ascribed to seedling recruitment. Other wetland plant species showed the existence of a guerrilla strategy role to escape from several factors exists [7, 11, 70], but none of these studies addressed the point that this strategy also becomes reflected at levels of clonal diversity and structure at spatiotemporal scales. We discuss each of these findings with respect to the hypotheses set and conclude by highlighting the implications for restoration and conservation activities.

In line with our first hypothesis, clonal growth traits of *C*. *papyrus* varied significantly between sedimentation regimes where phalanx strategy was more prevalent in HSR and guerrilla strategy in LSR. *C*. *papyrus* overcomes the adverse effect of HSR through production of denser ramets at the cost of a short culm height and smaller girth diameter, reflecting a consolidation strategy (phalanx) to exploit available resources created by sediment dynamics. Production of short and dense ramets with heterogeneous genets points to compensatory growth [71] in HSR. The mechanisms of compensatory growth in response to sediment and sand burial differ in plants [72, 73]. In contrast, in accordance with long spacers, longer culm height and relatively larger girth diameter, ramets under LSR showed a higher intermingling pattern which might reflect the foraging strategy [74], and corresponds to a guerrilla-type architecture. A shift from phalanx to guerrilla growth strategy has been confirmed as a principal strategy to escape from low to high sediment burial in *C*. *brevicuspis* [11], competition due to neighbour density in *Elymus lanceolatus* [7], sand burial in dune shrub *Hedysarum laeve* [75], sand accretion and nutrient availability in costal *Sporobolus virginicus* [76] and under conditions of different nutrient supplies in *Leymus secalinus* [70]. Our results from papyrus under natural conditions revealed a trade-off in clonal growth strategy from LSR to HSR that was the opposite of reports based on glasshouse and pot experiments [11, 70]. Clonal plants thus may have developed diverse clonal growth traits that underpin their adaptive strategies [7, 24, 25].

The clonal structure and diversity of *C*. *papyrus* revealed temporal and spatial fluctuation of genets. Those sites that experienced high sedimentation dynamics were more genotypically diverse in terms of clonal richness, Pareto distribution index and number of distinct genotypes, than were sites in LSR. The significant clonal subrange, *Dc* and the occurrence of few genets spatially restricted to either HSR or LSR reflect clonal sorting. The size and spatial distribution of genets determine demographic and sorting patterns, and have been found to be a function of trade-offs between clonal growth and sexual reproduction [77]. Like various measures of clonal growth (high ramet density, long spacer length and short culm) on *C*. *papyrus* genets under HSR, low *Dc*, *CR* and clone size related to a phalanx-type architecture. In addition, a skewed clone size distribution of *C*. *papyrus* implies dynamics in demographic processes at different stages as a response to sedimentation, which has also been reported for populations of the clonal seagrass, *Cymodocea nodosa* [69]. The latter only accounted for a difference at ramet level and genet level analysis associated with the reproductive modes, and did not include factors that could drive the demographic patterns in each population: yet historical contingencies during colonization are critical to understand factors driving genet distribution. A high *Dc* and low contemporary ramet densit**y** in LSR might also be attributed to the influence of asymmetrical competition between clones of different sizes, which amplifies loss of genets and decrease in genotypic diversity [78]. Under a relatively stable sediment regime (LSR), the founder effect of a few dominant clones may have shaped clonal structure through selection of superior clones and clonal integration [28, 79, 80]. According to the "general-purpose hypothesis", persistence and resilience are common features of large clones [79, 81]. However, overall, our results on papyrus do not support this hypothesis, because of the significant genet turnover and high clonal richness observed in sites with HSR.

Despite the fact that our data are limited to two sampling periods, the change in clonal composition, richness and growth strategies suggests the potential of an “allee effect”. Such a phenomenon has also been proposed for stand establishment of the clonal macrophyte, *Phragmites australis* [82]. The capacity of a species to colonize, establish and maintain its population in a dynamic environment is a function of dispersal potential. Individuals with identical MLGs distributed across the swamps studied have supported long-distance clonal dispersal within the same lake, as reported for spatially isolated *C*. *papyrus* populations [83], and selective advantages that could facilitate the clonal spread [84]. While the latter is more prominent within the same swamp area it may be less likely to occur in Lake Tana across such geographically distant swamps (68 km apart).

Papyrus populations revealed a wide range of clonal richness, from low (with very few genets) to high (with many genets) levels across LSR to HSR, respectively. This discrepancy in the number of genets explicitly highlights how the relative contribution of sexual and asexual reproductive modes dictates distribution and recruitment patterns of genets [17] in response to sedimentation. Initial seedling recruitment (ISR) and repeated seedling recruitment (RSR) are the two contrasting dynamic life history traits governing the growth of clonal plants [18]. Our results are in full agreement with a previous study that has provided evidence for seedling recruitment differences in *C*. *papyrus* populations along drawdown and inundated regimes at Lake Naivasha, Kenya [85]. High genet turnover ensuring an increased number of new genets in plots under HSR did reflect the central role of sexual reproduction and a RSR strategy. Despite high ramet density in these plots, sediment dynamics open windows for seedling recruitment and induce chaotic distribution of genets. Microsites created during these sediment dynamics influence the earliest stage of seedling emergence [86]. In contrast, the establishment of a few but dominant clones, coupled with maximum CR and longer spacer length in LSR revealed that an ISR model of *C*. *papyrus* was in place. In such a relatively stable regime, the presence of a cohort of a few large genets would show differential spatial spread (longer spacer length) and intragenet competition at the early stage of colonization [19]. Generally, our results showed that high sedimentation shifts the balance between seedling recruitment strategies (from ISR to RSR) and between the reproductive modes (from asexual to sexual). This, allows us a glimpse of the ultimate effect of environmental factors in driving microevolutionary forces [16] by influencing seedling establishment, and illuminates the comparative significance of reproduction modes [87, 88].

The resilience of wetlands and their keystone clonal plants have been reported as challenging, due to the spatial dynamics of genets that can be decoupled to allow contraction and expansion in response to biotic and abiotic factors [89]. However, our findings have twofold implications for resilience through habitat protection and restoration of *C*. *papyrus* populations. Severe land degradation in the upper catchments of the Lake Tana basin, for instance, is a far-reaching problem that led to lake over-sedimentation [27, 44]. However, with the premise that papyrus also controls sediment loads and provides socio-ecological services [32, 34, 38, 42], revegetation of sedimentation-prone sites using this species would lessen over-sedimentation and promote restoration in a broader context. To do this, selection of individuals of genets (genotypes) that withstand sediment dynamics and temporally persist (such as those noted in HSR), and sites potentially suitable for creating stepping stones between threatened populations [90], have to be the priority for mitigation of over-sedimentation along a lake margin. For sustenance of the studied papyrus swamps, preventive measures have to counteract anthropogenic pressures encompassing sand-mining and drainage of swamps for agricultural purposes [27]: these have negative impacts on the substrate, affecting for clonal growth and the recruitment of new genets. Our findings suggest that wetland conservation and restoration of keystone clonal plants should consider the spatiotemporal dynamics of ramets and genets that shape the level of genotypic diversity and determine the shift in clonal growth strategies for both short-term responses and long-term evolutionary potential.

In summary, we observed that the paper reed (*C*. *papyrus*) was able to adapt to sediment regimes by shifting clonal growth strategy from guerrilla (in LSR) to phalanx (in HSR). The threshold level of sedimentation that limits seedling recruitment and clonal dynamics has not been established for *C*. *papyrus*, but our spatial and temporal observations highlight that HSR enhances clonal richness owing to RSR and genets turnover. HSR creates a heterogeneous microenvironment that can be occupied by coexisting multiple genotypes, and this could have implications for population dynamics and resilience in the long term. Finally, restoration and conservation of papyrus wetlands from over-sedimentation, using clonal plants, should also incorporate information about spatiotemporal dynamics in clonal growth strategy, and in clonal diversity and structure.

## Supporting information

S1 Fig Clonal growth characteristics; ramet density (i) and spacer length (ii) of *Cyperus papyrus* under HSR and LSR observed for 2014 and 2016. The values (mean ± SE) recorded for 18 quadrates within each plot collated over the three swamps. Different letters indicate significant difference between years within a sedimentation regime and ** significant difference between sedimentation regime regardless of years (P < 0.05).
(TIF)

S2 Fig Maximum number of distinct genotypes that adequately reached the number of markers used, thereby allowing the accurate estimation of clonal diversity of *C*. *papyrus*. (TIF)

S3 Fig Frequency distribution of the pairwise number of alleles differences between MLGs for sample of *C*. *papyrus*. (TIF)

S1 Table Genotypic data of *C*. *papyrus* from three papyrus swamps in Lake Tana under two sedimentation regimes over two years. (XLS)

S1 Protocol Table: Multiplex PCR protocol for *C*. *papyrus*. (XLS)

S1 Text Copyright pemission granted to use the figure as shapefile. (PDF)

## References

[1] Vallejo-Marin M, Dorken E, Barrett SCH (2010) The ecological and evolutionary consequences of clonality for plant mating. Annual Review of Ecology Evolution Systematic 41:193–213.

[2] Cornelissen JHC, Song Y, Yu F, Dong M (2014) Plant traits and ecosystem effects of clonality: a new research agenda Annals of Botany 114: 369–376. doi: 10.1093/aob/mcu113 24948670 10.1093/aob/mcu113 PMC4111380

[3] Louapre P, Bittebiere AK, Clement B, Pierre JS, Mony C (2012) How Past and Present Influence the Foraging of Clonal Plants? PLoS ONE 7(6): e38288 doi: 10.1371/journal.pone.0038288 22675539 10.1371/journal.pone.0038288 PMC3365891

[4] Kui L, Li F, Moore G, West J (2013) Can the riparian invader, *Arundo donax*, benefit from clonal integration? Weed Research 53: 370–377.

[5] Ye D, Hu Y, Song M, Pan X, Xie X, Liu G et al (2014) Clonality–climate relationships along altitudinal gradient across China: adaptation of clonality to environments. PLoS ONE 9: e94009 doi: 10.1371/journal.pone.0094009 24709992 10.1371/journal.pone.0094009 PMC3977992

[6] Eckert GC (2001) The loss of sex in clonal plants. Evolutionary Ecology 15: 501–520.

[7] Li F, Xie Y, Zhu L, Jiang L, Chen X, Pan B, et al (2015) Changed Clonal Growth Form Induced by Sand Burial Facilitates the Acclimation of *Carex brevicuspis* to Competition. PLoS ONE 10 (3): e0121270 doi: 10.1371/journal.pone.0121270 25822734 10.1371/journal.pone.0121270 PMC4379005

[8] Thouvenot L, Haury J, Thiebaut G (2013) Seasonal plasticity of *Ludwigia grandiflora* under light and water depth gradients: an outdoor mesocosm experiment. Flora 208:430–437.

[9] Huang Y, Song Y, Li G, Drake PL, Zheng W, Li Z et al (2015) Morphological and structural plasticity of grassland species in response to a gradient in saline-sodic soils. Plant Biology 17: 1187–1195. doi: 10.1111/plb.12368 26177120 10.1111/plb.12368

[10] Yu FH, Roiloa SR, Alpert P (2016) Editorial: Global change, clonal growth, and biological invasions by Plants. Frontiers Plant Science 7:1467. 10.3389/fpls.2016.01467 PMC5040703 27746798

[11] Chen XS, Xie YH, Deng ZM, Li F, Houa ZY (2011) A change from phalanx to guerrilla growth form is an effective strategy to acclimate to sedimentation in a wetland sedge species *Carex brevicuspis* (Cyperaceae). Flora 206: 347–350.

[12] Humphrey LD, Pyke DA (2001) Ramet spacing of *Elymus lanceolatus* (thickspike wheatgrass) in response to neighbor density. Canadian Journal of Botany 79: 1122–1126.

[13] Tamm A, Kull K, Sammul M (2002) Classifying clonal growth forms based on vegetative mobility and ramet longevity: a whole community analysis. Evolutionary Ecology 15:383–401.

[14] Araki K, Shimatani K, Ohara M (2007) Floral distribution, clonal structure, and their effects on pollination success in a self-incompatible *Convallaria keiskei* population in northern Japan. Plant Ecology 189: 175–186.

[15] Riginos C, Liggins L (2013) Seascape genetics: populations, individuals, and genes marooned and adrift. Geography Compass 7: 197–216.

[16] Nanninga GB, Saenz-agudelo P, Manica A, Berumen ML (2014) Environmental gradients predict the genetic population structure of a coral reef fish in the Red Sea. Molecular Ecology 23:591–602. doi: 10.1111/mec.12623 24320929 10.1111/mec.12623

[17] Binks RM, Millar MA, Byrne M (2015) Not all rare species are the same: contrasting patterns of genetic diversity and population structure in two narrow-range endemic sedges. Biological Journal of the Linnean Society 114: 873–886.

[18] Eriksson O (1993) Dynamics of genets in clonal plants. Trends in Ecology Evolution 8: 313–316. doi: 10.1016/0169-5347(93)90237-J 21236180 10.1016/0169-5347(93)90237-J

[19] Becheler R, Benkara E, Moalic Y, Hily C, Arnaud-Haond S (2014) Scaling of processes shaping the clonal dynamics and genetic mosaic of seagrasses through temporal genetic monitoring. Heredity 112: 114–121. doi: 10.1038/hdy.2013.82 24022498 10.1038/hdy.2013.82 PMC3907096

[20] Alberto F, Massa S, Manent P, Diaz-Almela E, Arnaud-Haond S, Duarte CM et al (2008) Genetic differentiation and secondary contact zone in the seagrass *Cymodocea nodosa* across the Mediterranean-Atlantic transition region. Journal Biogeography 35: 1279–1294.

[21] Macreadie PI, York PH, Sherman CDH (2014). Resilience of *Zostera muelleri* seagrass to small-scale disturbances: The relative importance of asexual versus sexual recovery. Ecology and Evolution 4: 450–461. doi: 10.1002/ece3.933 24634729 10.1002/ece3.933 PMC3936391

[22] Maun MA (1998) Adaptation of plants to burial in coastal sand dunes. Canadian Journal of Botany 76: 713–738.

[23] Pan Y, Xie YH, Deng ZM, Tang Y, Pan DD (2014) High water level impedes the adaptation of *Polygonum hydropiper* to deep burial: responses of biomass allocation ad root morphology. Scientific report 4:5612 doi: 10.1038/srep05612 . 25002329 10.1038/srep05612 PMC4085590

[24] Mou XJ, Sun ZG (2011) Effects of sediment burial disturbance on seedling emergence and growth of *Suaeda salsa* in the tidal wetlands *o*f the Yellow River estuary. Journal of Experimental Marine Biology and Ecology 409:99–106.

[25] Chen XS, Deng ZM, Xie YH, Li F, Hou ZY, Li X et al (2014) Effects of sediment burial disturbance on the vegetative propagation of *Phalaris arundinacea* with different shoot statues. Aquatic Ecology 48: 409–416.

[26] Arnaud-Haond S, Marba N, Diaz-Almela E, Serrao EA, Duarte CM (2010) Comparative analysis of stability-genetic diversity in seagrass (*Posidonia oceanica*) meadows yields unexpected results. Estuaries and Coasts 33: 878–889.

[27] Wondie A (2010) Improving management of shoreline and riparian wetland ecosystems: the case of Lake Tana catchment. Ecohydrology and Hydrobiology 10:123–132.

[28] Jahnke M, Olsen LJ, Procaccini G (2015) A meta-analysis reveals a positive correlation between genetic diversity metrics and environmental status in the long-lived seagrass *Posidonia oceanica*. Molecular Ecology 24: 2336–2348. doi: 10.1111/mec.13174 25819368 10.1111/mec.13174

[29] Massa SI, Paulino CM, Serrao EA, Duarte CM, Arnaud-Haond S (2013) Entangled effects of allelic and clonal (genotypic) richness in the resistance and resilience of experimental populations of the seagrass *Zostera noltii* to diatom invasion. Ecology 13:39 doi: 10.1186/1472-6785-13-39 24152760 10.1186/1472-6785-13-39 PMC3818440

[30] Ehlers A, Worm B, Reusch TBH (2008) Importance of genetic diversity in eelgrass *Zostera marina* for its resilience to global warming. Marine Ecology Progress Series 355:1–7.

[31] Nevo E (2001) Evolution of genome-phenome diversity under environmental stress. Proceedings of the National Academy of Sciences, USA, 98, 6233–6240. 10.1073/pnas.101109298 PMC33451 11371642

[32] Mitsch WJ, Nahlik AM, Wolski P, Bernal B, Zhang L, Ramberg L (2010) Tropical wetlands: seasonal hydrologic pulsing carbon sequestration, and methane emissions. Wetlands Ecology Management 18:573–586.

[33] Mnaya B, Wolanski E (2002) Water circulation and fish larvae recruitment in papyrus wetlands, Rubondo Island, Lake Victoria. Wetland Ecology and Management 10: 133–143.

[34] Maclean IMD, Boar RR, Lugo C (2011) A review of the relative merits of conserving, using, or draining Papyrus Swamps. Environmental Management 47:218–229. doi: 10.1007/s00267-010-9592-1 21191792 10.1007/s00267-010-9592-1

[35] Kansiime F, Saunders M, Loiselle S (2007) Functioning and dynamics of wetland vegetation of Lake Victoria: an overview. Wetlands Ecology and Management 15: 443–451.

[36] Azzaa NGT, Kansiime F, Nalubega M, Denny P (2002) Differential permeability of papyrus and *Miscanthidium* root mats in Nakivubo swamp, Uganda. Aquatic Botany 67: 169–178.

[37] Owino AO, Ryan PG (2007) Recent papyrus swamp loss and conservation implication in Western Kenya. Wetlands Ecology and management 15: 1–12.

[38] Harper DM, Morrison EHJ, Macharia MM, Upton C (2011) Lake Naivasha, Kenya: ecology, society and future. Freshwater Reviews 4:89–114.

[39] Morrison JHE, Harper DM (2009) Ecohydrological principles to underpin the restoration of *Cyperus papyrus* at Lake Naivasha, Kenya. Ecohydrology and Hydrobiology 9: 83–97.

[40] Kyambadde J, Kansiime F, Gumaelius L, Dalhammar G (2004) A comparative study of *Cyperus papyrus* and *Miscanthidium violaceum*-based constructed wetlands for wastewater treatment in a tropical climate. Water Research 38:475–485. doi: 10.1016/j.watres.2003.10.008 14675660 10.1016/j.watres.2003.10.008

[41] Ryken N, Vanmaerke M, Wanyama J, Isabirye M, Vanonckelen S, Deckers J et al (2015) Impact of papyrus wetland encroachment on spatial and temporal variabilities of stream flow and sediment export from wet tropical catchments. Science of the Total Environment 511: 756–766. doi: 10.1016/j.scitotenv.2014.12.048 25617700 10.1016/j.scitotenv.2014.12.048

[42] Gaudet J. Papyrus: The Plant that Changed the World-From Ancient Egypt to Today’s Water Wars. New York: Pegasus Books L.L.C; 2014.

[43] Setegne SG, Srinivasan R, Dargahi B (2008) Hydrological modelling in the Lake Tana basin, Ethiopia using SWAT model. The Open Hydrology Journal 2: 24–40.

[44] Setegne SG, Srinivasan R, Melesse AM, Dargahi B (2010) SWAT model application and prediction uncertainty analysis in the Lake Tana Basin, Ethiopia, Hydrological Process 24:357–367.

[45] Friess DA, Krauss KW, Horstman EM, Balke T, Bouma TJ, Galli D et al (2012) Are all intertidal wetlands created equal? Bottlenecks, thresholds and knowledge gaps to mangrove and saltmarsh ecosystems. Biological Reviews 87: 346–366. doi: 10.1111/j.1469-185X.2011.00198.x 21923637 10.1111/j.1469-185X.2011.00198.x

[46] Chen X, Cao C, Deng Z, Xie Y, Li F, Hou Z et al (2015) Assessment of Regeneration Potential in the Clonal *Macrophyte Miscanthus* sacchariflorus (Poaceae) after Burial Disturbance Based on Bud Bank Size and Sprouting Capacity. PLoS ONE 10 (3): e0120846 doi: 10.1371/journal.pone.0120846 25785628 10.1371/journal.pone.0120846 PMC4365040

[47] Friis I, Demissew S, van Breugel P. Atlas of the Potential Vegetation of Ethiopia. Copenhagen: The Royal Danish Academy of Sciences and letters; 2010.

[48] Lamb HF, Bates CR, Coombes PV, Marshall MH, Umer M, Davies SJ et al (2007) Late Pleistocene desiccation of Lake Tana, source of the Blue Nile. Geophysical Research 8: 161–174.

[49] Hoyle M, James M (2005) Global warming, human population pressure and viability of the world’s smallest butterfly. Conservation Biology 19: 1113–1124.

[50] Minale AS, Rao KK (2011) Hydrological dynamics and human impact on ecosystems of Lake Tana, North-western Ethiopia. Ethiopian Journal of Environmental Studies and Management 4: 56–63.

[51] Kebede S, Travi Y, Alemayehu T, Marc V (2006) Water balance of Lake Tana and its sensitivity to fluctuations in rainfall, Blue Nile basin, Ethiopia. Journal of Hydrology 316: 233–247.

[52] Dargahi B, Setegn SG (2011). Combined 3D hydrodynamic and watershed modelling of Lake Tana, Ethiopia. Journal of Hydrology 398: 44–64.

[53] Tilahun SA, Guzman CD, Zegeye A, Ayana EK, Collick AM. Spatial and temporal patterns of soil erosion in the semi-humid Ethiopian highlands: A case study of Debre Mawi watershed In: Meless AM, Abtew W, Setegn SG, editors. Nile River Basin: Ecohydrological challenges, climate change and cydropolitics. Springer International Publishing; 2014pp. 149–163.

[54] Vijverberg J, Sibbing FA, Dejen E. Lake Tana: Source of the Blue Nile In: Dumont HJ, editor The Nile: Origin, Environments, Limnology and Human Use. Dordrecht: Springer Netherlands; 2009 pp. 163–192.

[55] Dessie M, Verhoest NEC, Admasu T, Pauwels VRN., Poesen J, Adgo E et al (2014) Effects of the floodplain on river discharge into Lake Tana (Ethiopia). Journal of Hydrology 519: 699–710.

[56] Poppe L, Frankl A, Poesen J, Admasu T, Dessie M, Adgo E et al (2013) Geomorphology of the Lake Tana basin, Ethiopia. Journal of Maps 9:431–437.

[57] Chebud YA, Melesse AM (2009) Modelling lake stage and water balance of Lake Tana, Ethiopia. Hydrological Processes 23: 3534–3544.

[58] Triest L, Sierens T, Terer T (2013) *Cyperus papyrus*: permanent genetic resources added to molecular ecology resources database 1 December 2012–31 January 2013. Molecular Ecology Resources 13: 546–549. 23521844 10.1111/1755-0998.12095

[59] Meglecz E, Costedoat C, Dubut V, Gilles A, Malausa T, Pech N et al (2010) QDD: a user friendly program to select microsatellite markers and design primers from large sequencing projects. Bioinformatics 26: 403–404. doi: 10.1093/bioinformatics/btp670 20007741 10.1093/bioinformatics/btp670

[60] Rozen S, Skaletsky H. Primer3 on the world wide web for general users and for biologist programmers In: Krawetz S, Misener S, editors. Bioinformatics methods and protocols: methods in molecular biology. Totowa, NJ: Humana Press; 2000 pp. 365–386. 10.1385/1-59259-192-2:365 10547847

[61] Holleley CE, Geerts PG (2009) Multiplex Manager 1.0: a cross platform computer program that plans and optimizes multiplex PCR BioTechniques 46:511–517. doi: 10.2144/000113156 19594450 10.2144/000113156

[62] Peakall R, Smouse PE (2006) GENALEX 6: genetic analysis in Excel. Population genetic software for teaching and research. Molecular Ecology Notes 6: 288–295. 10.1093/bioinformatics/bts460 PMC3463245 22820204

[63] Goude J. FSTAT Software, v. 2.9.3.2. 2002. http://www2.unil.ch/popgen/softwares/fstat.htm.

[64] van Oosterhout C, Hutchinson WF, Wills DP, Shipley P (2004) Micro-checker: Software for identifying and correcting genotyping errors in microsatellite data. Molecular Ecology Notes 4: 535–538.

[65] Arnaud-Haond S, Belkhir K (2007) GENCLONE: A computer program to analyse genotypic data, test for clonality and describe spatial clonal organization. Molecular Ecology Notes 7: 15–17.

[66] Dorken ME, Eckert CG (2001) Severely reduced sexual reproduction in northern populations of a clonal plant, *Decodon verticillatus* (Lythraceae). Journal of Ecology 83: 339–350.

[67] Arnaud-Haond S, Duarte CM, Alberto F, Serrao EA (2007) Standardizing methods to address clonality in population studies. Molecular Ecology 16: 5115–5139. doi: 10.1111/j.1365-294X.2007.03535.x 17944846 10.1111/j.1365-294X.2007.03535.x

[68] Ohsako T (2010) Clonal and spatial genetic structure within populations of a coastal plant, *Carex kobomugi* (Cyperaceae). American Journal of Botany 97: 458–470. doi: 10.3732/ajb.0900262 21622409 10.3732/ajb.0900262

[69] Alberto F, Gouveia L, Arnaud-Haond S, Perez-Llorens JL, Duarte CM, Serrao EA (2005) Within population spatial genetic structure, neighbourhood size and clonal sub-range in the seagrass *Cymodocea nodosa*. Molecular Ecology 14: 2669–2681. doi: 10.1111/j.1365-294X.2005.02640.x 16029469 10.1111/j.1365-294X.2005.02640.x

[70] Ye XH, Yu FH, Dong M (2006). A trade-off between guerrilla and phalanx growth forms in *Leymus secalinus* under different nutrient supplies. Annals of Botany 98: 187–191. doi: 10.1093/aob/mcl086 16687430 10.1093/aob/mcl086 PMC2803537

[71] Veeneklaas RM, Bockelmann AC, Reusch TBH, Bakker JP (2011) Effect of grazing and mowing on the clonal structure of *Elytrigia atherica*: a long-term study of abandoned and managed sites. Preslia 83: 455–470.

[72] Gilbert ME, Pammenter NW, Riofey BS (2008) The growth responses of coastal dune species are determined by nutrient limitation and sand burial. Oecologia 156: 69–178. 10.1007/s00442-008-0968-3 18246372

[73] Gilbert ME, Ripley BS (2010) Resolving the differences in plant burial responses. Austral Ecology 35: 53–59.

[74] Somme L, Mayer C, Jacquemart AL (2014) Multilevel spatial structure impacts on the pollination services of *Comarum palustre* (Rosaceae). PLoS ONE 9, e 99295. 10.1371/journal.pone.0099295 PMC4051681 24915450

[75] Li S, Yu F, Werger MJA, Dong M, During HJ, Zuidema PA (2015) Mobile dune fixation by a fast-growing clonal plant: a full life-cycle analysis. Scientific Report 5: 8935; doi: 10.1038/srep08935 25757743 10.1038/srep08935 PMC4355633

[76] Frosini S, Lardicci C, Balestri E (2012) Global change and response of coastal dune plants to the combined effects of increased sand accretion (Burial) and nutrient availability. PLoS ONE 7(10): e47561 doi: 10.1371/journal.pone.0047561 23077636 10.1371/journal.pone.0047561 PMC3471884

[77] Araki K, Shimatani K, Ohara A (2009) Dynamics of distribution and performance of ramets constructing genets: a demographic–genetic study in a clonal plant, *Convallaria keiskei*. Annals of Botany 104: 71–79. doi: 10.1093/aob/mcp092 19376781 10.1093/aob/mcp092 PMC2706722

[78] de Witte LC, Stocklin J (2010) Longevity of clonal plants: why it matters and how to measure it Annals of Botany 106: 859–870. doi: 10.1093/aob/mcq191 20880935 10.1093/aob/mcq191 PMC2990663

[79] Arnaud-Haond S, Duarte CM, ıaz-Almela DE, Marba N, Sintes T et al (2012) Implications of extreme life span in clonal organisms: millenary clones in meadows of the threatened seagrass *Posidonia oceanica*. PLoS ONE, 7, e30454 doi: 10.1371/journal.pone.0030454 22312426 10.1371/journal.pone.0030454 PMC3270012

[80] Kartzinel TR, Hamrick JL, Wang C, Bowsher AW, Quigley BG (2015) Heterogeneity of clonal patterns among patches of kudzu, *Pueraria montana* var. lobata, an invasive plant. Annals of Botany 116: 739–750. doi: 10.1093/aob/mcv117 26229064 10.1093/aob/mcv117 PMC4590328

[81] Lynch M (1984) Destabilizing hybridization, general-purpose genotypes and geographic parthenogenesis. Quarterly Review of Biology 59:257–290.

[82] Hazelton ELG, Mozdzer TJ, Burdick DM, Kettenring KM, Whigham DF (2014) *Phragmites australis* management in the United States: 40 years of methods and outcomes. AoB PLANTS 6: plu001; doi: 10.1093/aobpla/plu001 24790122 10.1093/aobpla/plu001 PMC4038441

[83] Triest L, Sierens T, Terer T (2014) Diversity and fine-scale spatial genetic structure of *Cyperus papyrus* populations in Lake Naivasha (Kenya) using microsatellite markers. Hydrobiologia 737:131–144.

[84] Grimsby JL, Tsirelson D, Gammon MA, Kesseli R (2007) Genetic diversity and clonal vs. sexual reproduction in Fallopia spp. (Polygonaceae). American Journal of Botany 94: 957–964. doi: 10.3732/ajb.94.6.957 21636464 10.3732/ajb.94.6.957

[85] Terer T, Muasya AM, Higgins S, Gaudet JJ, Triest L (2014) Importance of seedling recruitment for regeneration and maintaining genetic diversity of *Cyperus papyrus* during drawdown in Lake Naivasha, Kenya. Aquatic Botany 116: 93–102.

[86] Graae BJ, Ejrnaes R, Lang SI, Meineri E, Ibarra PT, Bruun HH (2011) Strong microsite control of seedling recruitment in tundra. Oecologia 166: 565–576. doi: 10.1007/s00442-010-1878-8 21170749 10.1007/s00442-010-1878-8 PMC3094527

[87] Lezberg AL, Halpern CB, Antos JA (2001) Clonal development of *Maiathemum dilitatum* in forests of differing age and structure. Canadian Journal of Botany 79: 1028–1038.

[88] Burgess SC, Treml E, Marshall DJ (2012) How do dispersal costs and habitat selection influence realized population connectivity? Ecology 93: 1378–1387. 22834378 10.1890/11-1656.1

[89] Mock KE, Rowe CA, Hooten MB, Dewoody J, Hipkins VD (2008) Clonal dynamics in western North American aspen (*Populus tremuloides*). Molecular Ecology 17: 4827–4844. doi: 10.1111/j.1365-294X.2008.03963.x 19140975 10.1111/j.1365-294X.2008.03963.x

[90] Perkol-Finkel S, Ferrario F, Nicotera V, Airoldi L (2012) Conservation challenges in urban seascapes: promoting the growth of threatened species on coastal infrastructures. Journal of Applied Ecology 49: 1457–1466.

