# Supplementary figures and images for: Clonal growth strategy, diversity and structure: A spatiotemporal response to sedimentation in tropical Cyperus papyrus swamps
Source: PLoS One. 2018 Jan 16;13(1):e0190810. doi: 10.1371/journal.pone.0190810 (PMC5770036; doi:10.1371/journal.pone.0190810)

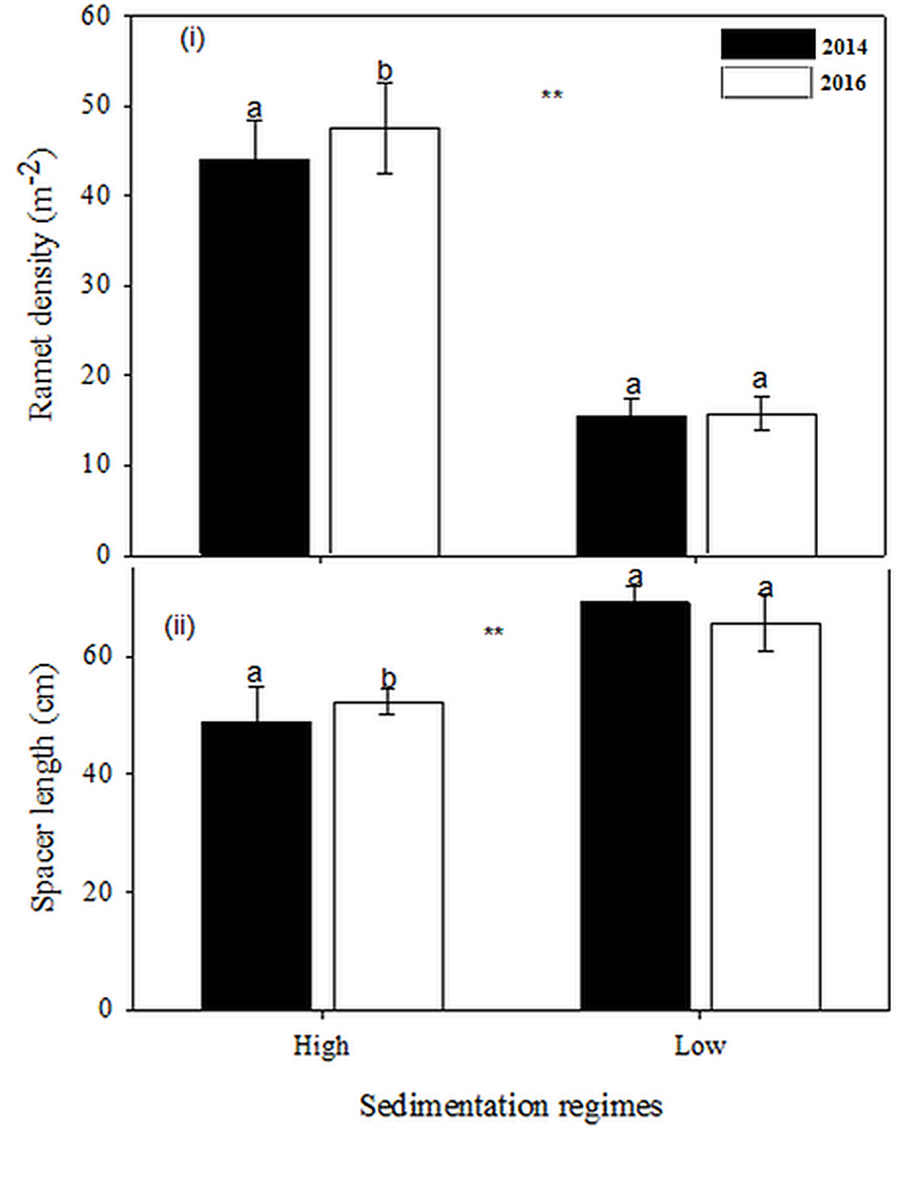

Supplement: S1 Fig — The values (mean ± SE) recorded for 18 quadrates within each plot collated over the three swamps. Different letters indicate significant difference between years within a sedimentation regime and ** significant difference between sedimentation regime regardless of years (P < 0.05). (TIF) [file pone.0190810.s001.tif]

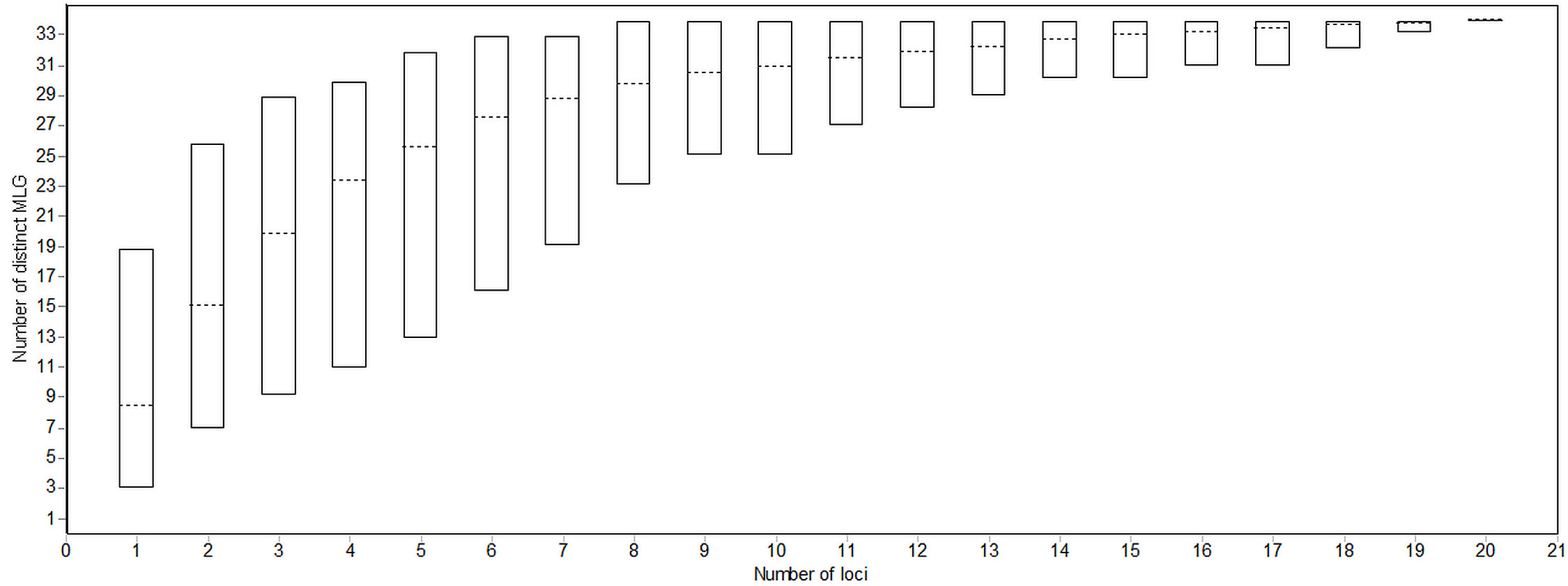

Supplement: S2 Fig — (TIF) [file pone.0190810.s002.tif]

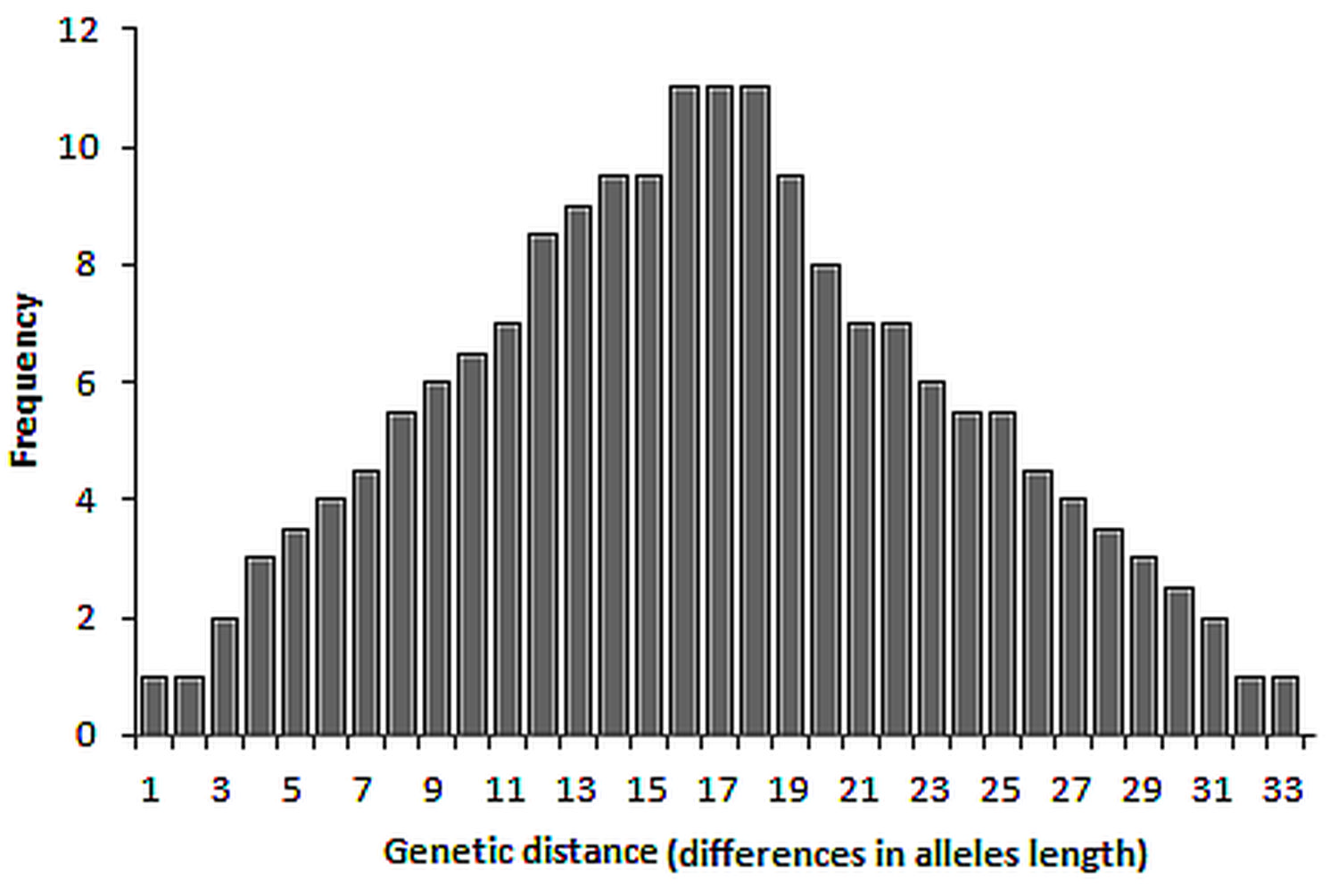

Supplement: S3 Fig — (TIF) [file pone.0190810.s003.tif]
